# Maximum lifespan and brain size in mammals are associated with gene family size expansion related to immune system functions

**DOI:** 10.1038/s41598-025-98786-3

**Published:** 2025-04-29

**Authors:** Huseyin Kilili, Benjamin Padilla-Morales, Atahualpa Castillo-Morales, Jimena Monzón-Sandoval, Karina Díaz-Barba, Paola Cornejo-Paramo, Orsolya Vincze, Mathieu Giraudeau, Stephen J. Bush, Zhidan Li, Lu Chen, Evangelos Mourkas, Sergio Ancona, Alejandro Gonzalez-Voyer, Diego Cortez, Humberto Gutierrez, Tamás Székely, Alín P. Acuña-Alonzo, Araxi O. Urrutia

**Affiliations:** 1 Milner Centre for Evolution, Department of Life Sciences, University of Bath, Bath, BA2 7AY UK; 2 Instituto de Ecología, Universidad Nacional Autónoma de México, Ciudad de México, 04510 Mexico; 3 UK Dementia Research Institute, Cardiff University, CF24 4HQ Cardiff, UK; 4 Licenciatura en Ciencias Genómicas, Universidad Nacional Autónoma de México, CP62210 Cuernavaca, Mexico; 5 Littoral, Environnement et Sociétés (LIENSs), UMR 7266 CNRS-La Rochelle Université, 2 Rue Olympe de Gouges, FR-17000 La Rochelle, France; 6 Institute of Aquatic Ecology, Centre for Ecological Research, 4026 Debrecen, Hungary; 7 Evolutionary Ecology Group, Hungarian Department of Biology and Ecology, Babeş-Bolyai University, 400006 Cluj-Napoca, Romania; 8 School of Automation Science and Engineering, Xi’an Jiaotong University, Xi’an, China; 9 Key Laboratory of Birth Defects and Related Diseases of Women and Children of MOE, Department of Laboratory Medicine, State Key Laboratory of Biotherapy, West China Second Hospital, Sichuan University, 610041 Chengdu, China; 10 Zoonosis Science Centre, Department of Medical Sciences, Uppsala University, Uppsala, Sweden; 11 Centro de Ciencias Genómicas, Universidad Nacional Autónoma de México, CP62210 Cuernavaca, México; 12 Instituto Nacional de Medicina Genomica, 14610 Ciudad de Mexico, Mexico; 13 Department of Evolutionary Zoology and Human Biology, University of Debrecen, Debrecen, Hungary

**Keywords:** Longevity, Ageing, Immunity, Gene duplication, Gene expression, Gene family size, Evolutionary genetics, Evolutionary biology, Comparative genomics, Genetics of the nervous system

## Abstract

Mammals exhibit an unusual variation in their maximum lifespan potential, measured as the longest recorded longevity of any individual in a species. Evidence suggests that lifespan increases follow expansion in brain size relative to body mass. Here, we found significant gene family size expansions associated with maximum lifespan potential and relative brain size but not in gestation time, age of sexual maturity, and body mass in 46 mammalian species. Extended lifespan is associated with expanding gene families enriched in immune system functions. Our results suggest an association between gene duplication in immune-related gene families and the evolution of longer lifespans in mammals. These findings explore the genomic features linked with the evolution of lifespan in mammals and its association with life story and morphological traits.

**Supplementary Information:**

The online version contains supplementary material available at 10.1038/s41598-025-98786-3.

## Introduction

Mammals exhibit high diversity in their maximum lifespan potential (MLSP, the age at death (longevity) of the longest-lived individual ever recorded in a species), ranging from less than a year in some shrew species to over a hundred years in humans and up to two hundred in bowhead whales^1^. Even in captivity lifespan variation persists, pointing to intrinsic biological factors limiting an individual’s longevity^2,3^. Unlike average lifespan, which reflects both intrinsic and extrinsic factors such as the risk of predation and resource availability, MLSP is assumed to reflect a species’ inherent longevity limit and is widely available used in comparative studies focused on life history trade-offs and the genomic determinants of longevity^4–10^.

Chiroptera (bats), the second-largest mammalian order, represent an important model to study longevity and ageing^11^. Bats exhibit extended lifespans relative to body size^12,13^, disease resistance to ageing-related illnesses such as cancer^14^, viral infections^15^. Similarly, genomic analyses of the bowhead whale (MLSP larger than 200 years) have identified changes in genes related to DNA repair, cell-cycle regulation, cancer and ageing^16^. Other long-lived mammals, such as elephants, show expansion in gene families associated with DNA repair and tumour suppression, including *TP53* ^17^.

MLSP is thought to reflect intrinsic differences in the molecular machinery governing the ability of organisms to cope with age-related cognitive and/or physical decline and vulnerability to disease^18–24^. Studies on the molecular basis of longevity, suggest that non-dividing differentiated cells (e.g., neurons) may be a limiting factor for longevity^25,26^. In humans, genes linked to post-mitotic cell longevity are enriched in pathways like cytoskeleton-dependent transport, tRNA metabolism, cell morphogenesis, and ribosome biogenesis^25,26^. These genes show reduced expression in neurodegenerative diseases and progeria, hinting at a protective role against ageing^25,26^. However, how these mechanisms have influenced MLSP evolution across species remains unclear.

Identifying the overarching genomic signatures associated with the evolution of MLSP can provide insights into the evolution of key life history traits and variations in longevity between individuals in a species. Comparative studies have linked MLSP variations to changes in gene expression profiles and differences at the genome sequence level, including protein rates of evolution and gene family size differences^27–29^. Genes associated with MLSP in these studies were enriched in DNA repair, defence response cell cycle and immunological process related terms^28^. Genes such as *PMS2* (DNA repair), *PNMA1* (cell fate determination), and *OGDHL* (ROS regulation) show positive correlation with MLSP across mammalian tissues. *BCL7B*, which inhibits carcinogenesis through Wnt pathway regulation, and *GATM*, associated with oxidative stress protection, are prominently linked to increased lifespan. These molecular signatures collectively enhance cellular maintenance and stress resistance mechanisms that appear critical for extended longevity^30^.

Gene family size evolution, driven by duplication and deletion events, has been suggested to play a key role in phenotypic evolution^31–34^. While many gene duplication events in some cases can result in pseudogenization and reversal to pre-duplication copy number, preserved duplicates can increase gene dosage^35,36^, or expand transcript and protein diversity^37^. Although overall gene number has remained stable over 800 million years of metazoan evolution, gene family expansions and contractions have phenotypic diversity^38–43^. Genome sequencing projects for long-lived mammals have uncovered gene duplication events, likely linked to increased longevity, such as in the bowhead whale^16^ and naked mole rat^44^.

Life history and morphological traits influencing MLSP have yet to be considered in comparative genomic and transcriptomic studies. Body mass presents a positive correlation with MLSP^45^, possibly because higher vulnerability of smaller species to predation compared to larger ones and the prioritisation of reproduction over self-maintenance to maximise fitness^46^. Another explanation is the negative correlation between metabolic rates with body mass in endothermic animals^47^, as accelerated metabolism in small species ultimately results in a faster accumulation of molecular damage^48,49^. Gestation time^23^ and age at sexual maturity^50^ also show a positive association with MLSP, reflecting potential developmental constraints linked to monotocous large-body mass mammals with long life spans^51^. Brain size relative to body mass is another key correlate, as large species tend to have large MLSP^52–54^, potentially due to higher behavioural complexity and survival strategies^55,56^.

It is also important considering that larger species have lower effective population sizes, which may weaken purifying selection across the genome^57^, influencing genomic features such as transposable element content, intron and intergenic region length^57–59^. A study examining over 100 bird species genomes found evidence of lower purifying selection in larger species^60^, though some evidence suggests it remains effective even in species with an small effective population^6^. Thus, any genomic feature associated with MLSP must be assessed to exclude the confounding effects of reduced purifying selection in long-lived species. No prior study examining genomic correlates of MSLP has considered this factor.

Despite the growing body of research on longevity and MLSP genomics and transcriptomics^61^, gene lists identified in individual studies are rarely directly compared with previous findings. Existing lists include genes associated with MLSP across species and those linked to longevity variation within species. Comparisons have shown little overlap, suggesting that molecular mechanisms underlying evolutionary MLSP changes may differ from those governing species longevity differences. Performing such comparisons would enhance the interpretation of the results obtained and assess how different aspects of genome evolution in line with MLSP, such as gene expression changes, protein rates of evolution, and gene family expansions, relate to one another and within species genome-wide association studies in human leading to a more integrated view of this key phenotype.

Furthermore, several biological processes have been proposed to contribute to ageing and longevity, including DNA repair^62^, apoptosis^63,64^, immunity, inflammation^65^, autophagy^66^, oxidative stress^67^ and epigenetic markers^68^. Interventions such as caloric restriction and compounds known as senolytics can modulate longevity, highlighting the importance of these pathways. Directly testing the enrichment of genes related to these processes could magnify our understanding of MLSP evolution.

Here, we use a comparative genomics approach to identify genomic signatures associated with the evolution of MLSP across mammals. We examine whether MLSP variations correlate with gene family sizes (of protein-coding genes) in 46 fully sequenced mammalian species, accounting for potential confounders such as body mass, brain size, and effective population size. Specifically, we consider the potential confounding effect of a generalised genome-wide gene family size increase in line with MLSP resulting from diminished purifying selection among longer-lived species. Furthermore, We conduct a genome-wide analysis not restricted to specific functional gene categories and analyse the overlap between MLSP-associated genes identified in this study with gene sets previously associated with human longevity and molecular processes relevant to ageing and longevity evolution.

## Results

### Significant association between MLSP and gene family expansion

**Fig. 1:**
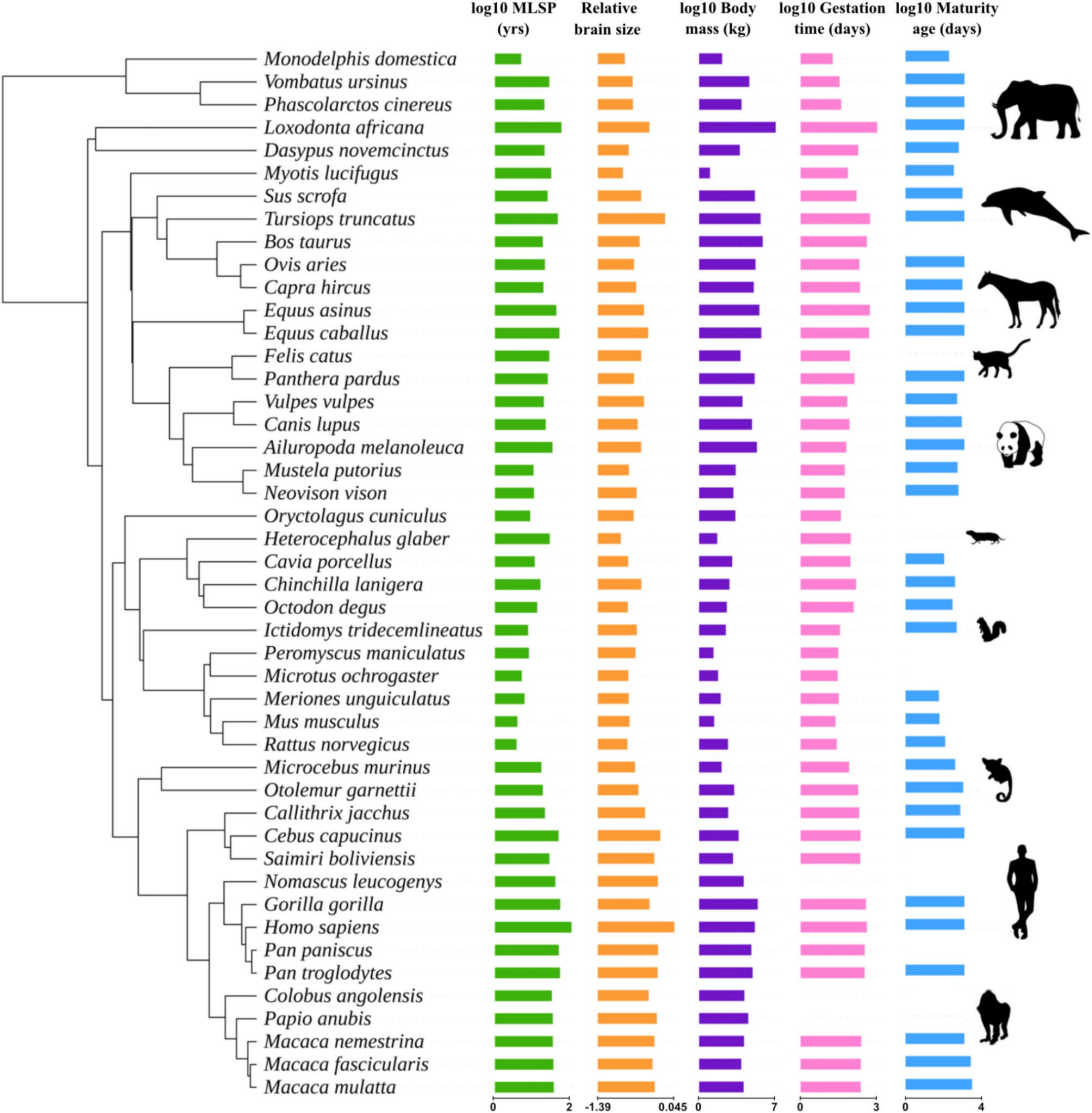
Phylogenetic distribution of life history and morphological traits (maximum lifespan potential, relative brain size, body mass, gestation time and age at sexual maturity) in mammals. A phylogenetic tree for 46 mammal species with fully sequenced genomes is shown. Bars show the relative value of each variable, including log10 values of maximum lifespan potential (MLSP, green), relative brain size (orange), body mass (purple), gestation time (pink), and age at sexual maturity (blue). See Supplementary Table 1 for raw data and silhouette species names.

**Fig. 2:**
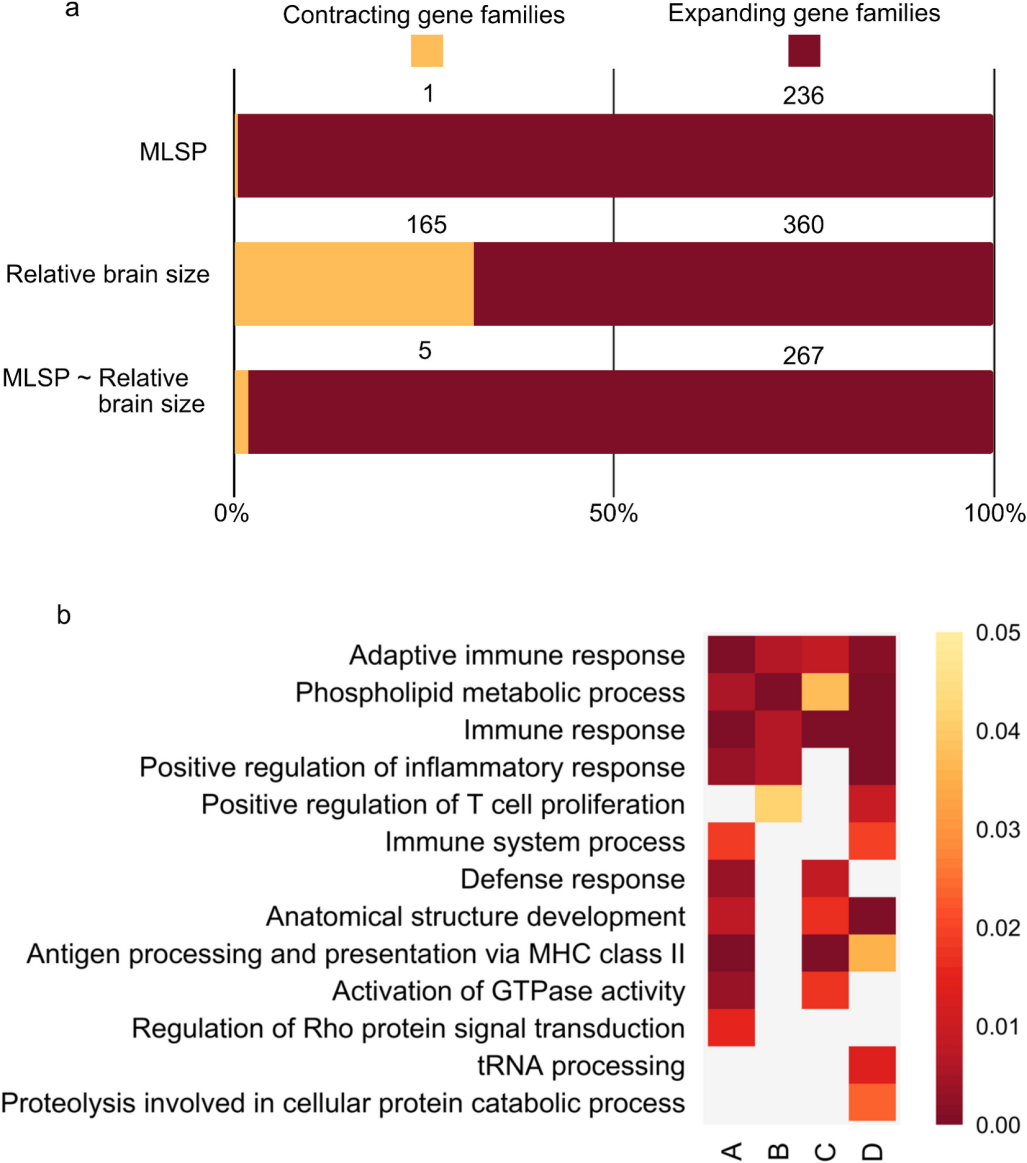
Significantly associated and functional annotation overrepresentation among gene families with size variations in mammals. Panel (**a**) shows the number of gene families expanding and contracting associated with MLSP; relative brain size; and MLSP corrected by relative brain size. (**b**) Gene ontology (GO) term enrichment analysis among the families significantly associated with several PGLS models. Coloured cells in each column represent significantly enriched GO categories after correction for multiple testing (Benjamini Hochberg). Colour intensity (towards purple) increases for smaller adjusted *p-values*. Individual columns show significantly enriched categories for (A) MLSP-associated families from PGLS model which has 46 species (*r*_MLSP−associated families_ > 0, *p* < 0.05, *n* = 236), (B) relative brain size-associated families from PGLS with 59 species (*r*_relative brain size−associated families_ > 0, *p* < 0.05, *n* = 360), (C) MLSP-associated families identified in 46 species in a PGLS model containing both MLSP and relative brain size (*r*_MLSP−associated families_ > 0, *p* < 0.05, *n* = 267), (D) relative brain size-associated families from PGLS model that contains MLSP and relative brain size (*r*_relative brain size−associated families_ > 0, *p* < 0.05, *n* = 184).

We conducted a phylogenetic generalised least-square (PGLS) analysis, corrected by Benjamini-Hochberg^69^, to analyse the association between gene family size (dependent variable) and MLSP (independent variable). A total of 4,136 gene families in 46 fully sequenced mammalian species were included in the analysis (Fig. 1; supplementary Table 1). Our analysis calculated 236 statistically significant MLSP-associated families under expansion (*p* < 0.05; effect sizes ranging from *r* = 0.43 to 0.60; Fig. 2a), while one presented significant contraction (*p* < 0.05; effect size of *r* = -0.49; Fig. 2a). It has been suggested that several gene features proliferate in genomes of species with smaller effective population sizes. To rule out a possible generalised increase in protein-coding gene number among species with higher MLSP, we tested the association between total protein-coding genes and MLSP. Our findings indicate no significant association (p *>* 0.05).

### Relative brain size, not body mass nor life history traits, influences gene family expansion associated with MLSP

We then tested whether body mass and relative brain size could explain the observed associations between gene family size and MLSP. In a set of 46 species with data available for all three variables, we observed a significant association between MLSP with both body mass (*r* = 0.60; *p <* 0.0001) and relative brain size (*r* = 0.70; *p <* 0.0001, Supplementary Fig. 1). Gene family size analysis found 360 expanding gene families and 165 contracting gene families associated with relative brain size after Benjamini-Hochberg correction (Fig. 2a). For body mass, our analysis did not find significant associations between MLSP and gene family size.

Subsequently, in another set of 42 and 31 species, we examined whether MLSP correlated with gestation time and age at sexual maturity, respectively. We detected positive correlations with MLSP (single-predictor PGLS models: MLSP vs. gestation time; *r* = 0.70; p *<* 0.0001 and MLSP vs. age at sexual maturity; *r* = 0.80; p *<* 0.0001; Supplementary Fig. 1). We did not find significant associations between the models analysing gene family size and gestation time or age at sexual maturity.

To identify gene families associated with MLSP after accounting for the effect of relative brain size, we ran a two-predictor PGLS regression model that included both MLSP and relative brain size. We found 267 gene families presenting expansion while five were undergoing contraction (Fig. 2a). The five gene families undergoing contraction include genes involved in biological processes, such as blood clotting^70^, transcription regulation^71^, cytoskeleton organisation^71^, cholesterol trafficking^71^, and protein hydrolysis^71^ (Supplementary Table 2). A total of 184 expanding gene families were associated with relative brain size. Of these, 161 gene families were significantly associated with both traits, consistent with a shared evolutionary path. The 267 gene families that present expansion include a total of 2061 genes. Henceforth, these will be referred to as MLSP-associated families and MLSP-associated genes, respectively. Similarly, the 184 gene families associated with relative brain size comprise 1673 genes. These will be referred to as relative brain size-associated gene families and hereafter referred to as relative brain size-associated genes.

Sensitivity test using a Leave-One-Out approach, assessing effect size and significant changes in the correlation structure indicate that most species have a negligible effect size (Cohen’s d < 0.2) and non-significant variations in *r* values (*p* > 0.05). However, *Heterocephalus glaber* (Cohen’s d = − 0.22, Wilcoxon: *p* < 0.002) and *Homo sapiens* (Cohen’s d = − 0.59, Wilcoxon: *p* > 0.05) exhibit larger effect size, while *Gorilla gorilla* (Cohen’s d = 0.13, Wilcoxon: *p* < 0.002) and *Loxodonta africana* (Cohen’s d = 0.13, Wilcoxon: *p* < 0.002) show small but significant effect (Supplementary data 1). This suggests that our observations might not be solely driven by one species but can still be influenced by taxa with extreme values.

### Immune system functional annotations are enriched among MLSP-associated and relative brain size-associated genes

MLSP-associated genes showed a significant overrepresentation of immune system-related functional annotations (Fig. 2b; supplementary Table 4). No functional enrichment analysis was conducted, given the small number of MLSP-associated contracting gene families (*n* = 5). Relative brain size-associated genes show similar functional enrichment patterns to MLSP-associated genes, with significantly overrepresented immune system functions. On the other hand, contracting relative brain size-associated gene families were not enriched in any functional category.

### MLSP-associated genes present higher gene expression and alternative splicing in the human

The observed gene family expansions associated with the evolution of MLSP may respond to selective pressures on gene dosage^35,36^ or selective pressures related to transcript diversity^37^. Thus, we next examined gene expression patterns and alternative splicing in MLSP-associated genes in humans. We found that MLSP-associated genes have a higher gene expression level and produce a higher number of unique transcripts compared to background genes (Wilcoxon rank sum test: *p* FPKM *<* 0.0001 and *p* for Transcript Number = 0.0022, respectively). No significant results were obtained when examining relative brain size-associated genes (Wilcoxon rank sum test: *p* FPKM *>* 0.05 and *p* Transcript Number *>* 0.05, respectively). However, since this analysis is based solely on human data, these results should be interpreted with caution, as gene expression and splicing patterns may differ across species and tissue types. Future studies incorporating cross-species transcriptomic data will be necessary to determine the broader evolutionary significance of these findings.

**Fig. 3:**
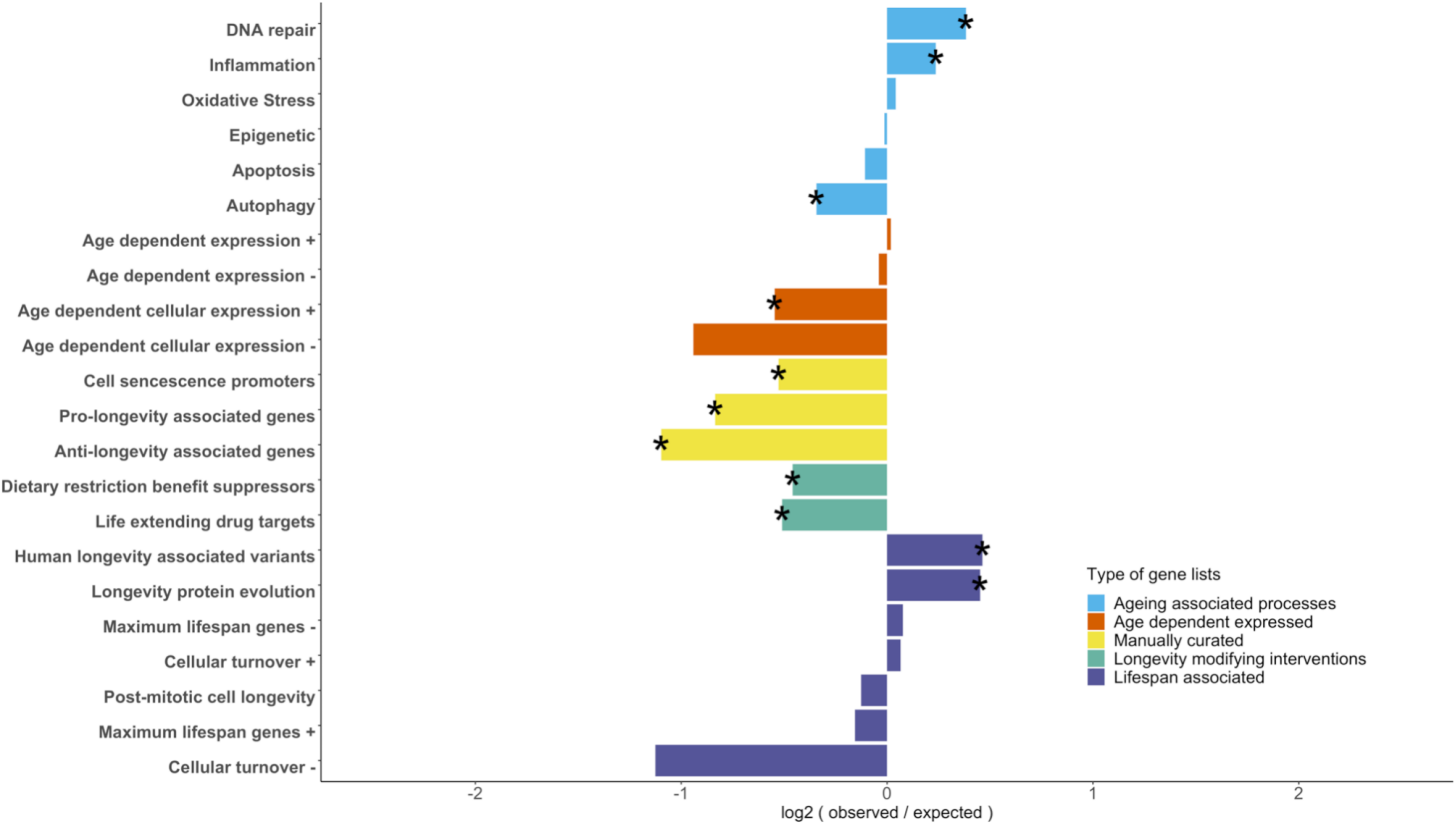
Longevity-associated databases gene enrichment among MLSP-associated genes. Enrichment analysis of longevity-related databases among MLSP-associated genes. Bar lengths show the log2 values of the observed number of genes from individual datasets divided by the expected number of genes under the null hypothesis. Bars are colour-coded according to the type of gene lists collected. Stars denote a significant deviation from the null under a two-tail chi-square test.

Genes previously associated with ageing and longevity are also enriched among MLSP-associated gene families. We assessed whether MLSP-associated genes significantly overlap with genes previously shown to (a) be associated with related molecular processes, (b) have age-dependent expression, (c) be manually curated associated with ageing or longevity, (d) targets of longevity modifying interventions and (e) be associated with lifespan and longevity within and between individuals and across species (Fig. 3). Among genes with functional annotations of molecular processes previously suggested to play important roles in ageing and longevity, MLSP-associated genes were significantly enriched in genes with DNA repair (*X*^2^ test *p* = 0.0028)^72^ and inflammation^73^ (*X*^2^ test *p* = 0.0002) functions with a significant underrepresentation of autophagy^74^ (*X*^2^ test *p* = 0.0075) related genes. No significant over or under-representation was observed for genes related to oxidative stress^73^, epigenetic markers^75^ and apoptosis^76^. Genes with age-dependent expression that increase in activity with age were found to be underrepresented among MLSP-associated genes for one database but not for a second one (“age-dependent cellular expression”^77^ and “age-dependent expression”)^27^. Genes that decrease their expression with age obtained from the same sources were not over or under-represented among MLSP-associated genes. Two sets of manually curated gene lists for cell senescence^29^ (*X*^2^ test *p* = 0.0024) and longevity^78,79^ were found to be significantly underrepresented among MLSP-associated genes. Similarly, significant underrepresentation among MLSP-associated genes was observed for genes that suppress the life-extending effects of caloric restriction^80^ (*X*^2^ test *p* = 0.0058) and targets of life-extending drugs (senolytics)^81–84^ (*X*^2^ test *p* = 0.0036). Among gene lists correlating with cell longevity, individual longevity and species lifespan, significant over-representation among MLSP-associated genes (*X*^2^ test p *<* 0.0001) was observed for genes with human centenarians-associated genetic variants^85^ and among genes with faster protein evolution in species with higher MLSP; no significant under of over-representation was observed for genes whose transcriptional profiles correlate with MLSP across mammalian species^86^ or those associated with postmitotic cellular longevity^25,26^.

To further understand the functional relevance of MLSP-associated genes and how they compare to gene sets from previous studies, we conducted a GO enrichment analysis. Finding highly significant (*p* < 0.01) enrichment of immune system-related (e.g. innate immune response, immune response, adaptive immune response, inflammatory response) and longevity-related (e.g. DNA repair, negative regulation of apoptotic process, autophagy, DNA damage response, regulation of apoptotic process) functions among the MLSP-associated genes and genes presented in previous studies (Supplementary Fig. 2). Apoptosis, senescence, Ei-associated, human longevity associated variants, life extending drug targets and MLSP-associated genes share several biological functional annotations. In contrast, genes previously associated with age-dependent cellular expression, age dependent expression, cellular turnover, dietary restriction benefit suppressors, DNA repair and post-mitotic cell longevity show none or minimal overlap with the other gene sets. These results suggest that while MLSP-associated genes share common pathways with some immune and longevity-related processes discovered in previous studies, other longevity-related genes may represent distinct mechanisms of lifespan regulation not shared among the species analysed in this study.

## Discussion

We identified 236 gene families showing significant positive correlations with maximum lifespan potential across the mammalian phylogeny. Genes in these families are enriched in immune system functional annotations and among genes previously associated with ageing and longevity. Unlike earlier studies, our work accounts for potential confounding effects of morphology variables and life history traits when exploring genomic signatures associated with lifespan evolution.

Our findings are unlikely to be explained by smaller effective population sizes among species with the highest MLSP. Among mammals, species with the highest MLSP also tend to be larger, which correlates inversely with effective population size^57–59^. However, we found no significant correlation between MLSP and total protein-coding gene number. Furthermore, body mass, which correlates with effective population size^87^, was not associated with gene family size increases.

We uncovered evidence of a shared molecular machinery associated with the evolution of MLSP and relative brain size, with 161 gene families significantly related to both phenotypes when included in the same models. This common genomic signature aligns with the established evolutionary relationship between lifespan and relative brain size in vertebrates^54,88^. Conversely, no associations were found with gestation time, age at sexual maturity, or body mass.

Gene ontology analysis of MLSP-associated families revealed consistent enrichment of genes involved in “immune response”, “defence response”, “adaptive immune response” and “antigen processing and presentation via MHC class II” processes. The immune system has multiple mechanisms that could support longer lifespan: removing senescent cells (critical for neuron maintenance)^89^ detecting and removing cells with uncontrolled cell division^90^infections^91^. These findings support the concept that the immune system plays an important role in determining lifespan and shaping its evolution across the animal kingdom, including humans^92–95^.

Interestingly, MLSP-associated families are significantly enriched in genes with human longevity-associated variants^85^. Functional enrichment analysis of these human longevity-associated genes^85^conducted in this study revealed immune system-related functions among the enriched gene ontology categories. While previous studies found little overlap between genes with MLSP-associated protein evolution rates and genes with longevity-associated variants in humans^9^, our enrichment comparing our MLSP-associated genes and multiple previous studies’ gene sets shows functional convergence. This convergence is particularly evident in immune system regulations and apoptotic processes, indicating that despite identifying different gene sets, these studies highlight common underlying biological functions. However, a more recent study examining gene expression patterns across tissues for species with different MLSP did find some genes in common when examining age-associated genes within species^10^, suggesting a degree of commonality on specific aspects of MLSP and within species variation in longevity. These results suggest an overlap between molecular mechanisms associated with lifespan evolution across species and those involved in longevity differences within a species. Future studies directly testing overlap between genes identified within and between species comparisons should establish the extent of the common molecular pathways explaining evolutionary patterns and between individual differences.

Among biological processes previously associated with ageing and longevity, we found that genes associated with DNA repair and inflammation are significantly overrepresented among MLSP-associated genes, while autophagy-associated genes are underrepresented. This is noteworthy as autophagy has been found to remain stable in longevity model species like the naked mole rat^96^. Our functional enrichment analysis further reinforces these associations, revealing that MLSP-associated genes share significant functional enrichment with previously identified longevity-related processes, particularly in DNA damage response, inflammation regulation and apoptotic processes, despite limited overlap in the specific genes identified.

However, we did check the overlap between our MLSP-associated gene set and the candidate gene list published in a previous study^16^, and these genes were significantly underrepresented among MLSP-associated genes. This suggests that this manually curated gene list is potentially capturing a different aspect of longevity-associated molecular mechanisms not involved in driving the evolution of MLSP.

Alternative splicing has long been proposed as an alternative mechanism for expanding transcript diversity in addition to gene duplication^37^. Past studies have suggested an inverted correlation between gene family size and alternative splicing^97–99^, suggesting that the two mechanisms are, to some extent, equivalent^100^. We observed that MLSP-associated genes are more highly expressed and more alternatively spliced than background genes. These results may reflect an expansion of both gene expression and alternative splicing among MLSP-associated genes in line with MLSP evolution. However, in-depth comparative studies examining gene expression and alternative splicing across different species are needed to assess the selective pressures behind gene family expansions in line with MLSP evolution.

Our functional analyses reveal a meaningful picture, demonstrating that while direct gene list overlaps between studies may be limited, there is substantial convergence at the functional level. MLSP-associated genes share significant functional annotations with several gene sets (including apoptosis, senescence, human longevity-associated variants, and life-extending drug targets), particularly in immune system response and longevity-related processes. This functional convergence suggests that while different studies may identify distinct gene sets, they often highlight the same biological pathways, reinforcing the importance of these processes in longevity determination across phylogenetic lineages. The minimal functional overlap observed with certain gene sets (age-dependent expression, dietary restriction benefit suppressors, post-mitotic cell longevity) further suggests that some longevity mechanisms may represent distinct evolutionary pathways not universally shared among the species analysed.

In this study, we use MLSP as a measure of the intrinsic limit of longevity for individuals in each species. While widely used in comparative genomic and transcriptomic studies, MLSP is not a perfect index. The longest-living individual in a species might reflect an outlier with unique mutations absent from the reference genome, and in species with limited longevity records, MLSP may approximate average lifespan rather than longevity potential. Furthermore, whether records are taken from wild or captive individuals may influence evolution inferences. As demography-based indexes based on mortality risk by age become available, a clearer picture of the genomic basis of lifespan will emerge^101^. However, given the differences in MLSP among the species included in our study, we consider it is unlikely that the above-mentioned limitations would significantly impact our results.

Our findings are consistent with a scenario where gene family expansions have contributed to the evolution of longer lifespans in mammals. However, the associative nature of this study prevents establishing causality. We cannot rule out the possibility that gene family size variations are a by-product of longer lifespan evolution driven by unknown molecular mechanisms, or that other genomic changes unrelated to gene family size variations drive both phenotypic changes in MLSP.

## Conclusions

In summary, our study conducts a comprehensive genome scan for signatures of gene family expansions in line with the evolution of longer maximum lifespans. Our results on gene family expansions associated with MLSP are robust, remaining significant even when accounting for potential confounding variables, including life story traits (gestation time, age at sexual maturity) and morphological traits (body mass). Notably, following earlier evidence of MLSP being driven by the evolution of larger brains, we find evidence for a strong common genomic signal in gene family expansions for both phenotypes. Thus, we propose that relative brain size must be considered in future studies investigating genomic signatures of MLSP. Interestingly, we find significant and consistent enrichment of immune system genes among MLSP-associated genes. Additionally, we find that MLSP-associated families significantly overlap those associated with human longevity, suggesting shared molecular mechanisms between the evolution of MLSP across species and the variation in longevity in humans.

## Materials and methods

### Gene family size annotations and genome completeness

Annotated gene families and fasta sequences encompassing 92 fully sequenced mammalian genomes were obtained from Ensembl^102^ using the *BiomaRt* R Package^103^ (*n* = 17,722). To exclude lower-quality and incomplete genomes, which could bias the assessment of gene family sizes, we used the Benchmarking Universal Single-Copy Ortholog (BUSCO) tool^104^ using the OrthoDB v10 database for mammals as benchmarking to assess genome completeness. A total of 46 species (supplementary Table 1) with genome completeness scores higher than 80% were considered further. Gene families with no variance in gene number across species were excluded from the analyses^34^. Gene families were also required to have at least three genes in one species. Finally, gene families must be present in at least 80% of species to rule out lineage-specific gene families. This resulted in 4,121 gene families^34^.

### Maximum lifespan potential, brain mass, body mass and relative brain size

Maximum lifespan potential (MLSP) and body mass (BM) estimates for 46 species (supplementary Table 1) with good-quality annotated genomes were obtained from the Animal Aging and Longevity Database build 14 ^79^. Brain mass measurements were also obtained for 46 species from various sources (supplementary Table 1). Brain mass and body mass estimates were used to obtain relative brain size values, which quantify brain mass controlling for the allometric effect of body size, by calculating the residuals of a log-log least squares linear regression of brain mass against body mass as applied by^34,54^ (see supplementary Tables 1 and references therein).

### Correlation of maximum lifespan potential with other traits

Correlations between phenotype traits were assessed using independent contrast correlations, which allow assessing relatedness between two variables after correction for phylogenetic relatedness^105^ using corrgram function in *R*.

### Phylogenetic regressions of gene family size with life history and morphological traits

Ultrametric phylogeny of the 46 mammalian species with available good-quality genome and phenotypic data was obtained from Ensembl^102^. Phylogenetic tree and trait data were visualised using iTOL^106^. We used phylogenetic generalised least square regressions to assess the strength of associations between gene family sizes and life history or morphological traits across species. To rule out associations between phenotypic parameters and variations in gene family size being explained by shared ancestry, a phylogenetically generalised least squares regression (PGLS)^107,108^ was used. Phylogenetically corrected regressions were performed using the “nlme” R package^109^, assuming a Brownian motion model of evolution and using the maximum likelihood method. Benjamini-Hochberg correction for multiple testing was implemented to identify gene families associated with the phenotypic traits examined^69^. Additionally, to evaluate the robustness of our findings and quantify the influence of individual species we conducted a Leave-One-Out sensitivity test by iteratively removing each species and re-estimating the two-predictor PGLS model. For each iteration we used statistically significant *t* values to calculate Cohen’s d, using “effsize” R package^110^, to assess the effect size of each removed species. Later we conducted a Wilcoxon rank-sum test, using “stats” R package^111^, to detect significant deviations of *r* values from the original results.

### Gene ontology term enrichment analysis

Biological process gene ontology (GO) functional terms annotations for each gene for each species were obtained from the Gene Ontology Consortium database^112^. GO terms were linked to a family whenever that term was assigned to any gene in the family in any of the 46 sequenced mammalian species available in Ensembl^102^. To prioritise GO terms with high association with gene families and increase the potential functional significance, we excluded GO terms annotated to less than 50 gene families from the analysis^68^. Enrichment of GO categories among the set of gene families associated with each trait of interest was carried out by measuring the proportion of families assigned to each GO term within the analysed set of gene families and comparing it with the proportion of gene families associated with each GO term in 1,000 equally sized samples of randomly chosen gene families from the background set. The mean and standard deviation of GO term representation measured in each 1000 random samples were taken to determine the corresponding p-values for each GO term using Z-scores with Benjamini-Hochberg correction for multiple testing, as implemented in^34^.

### Ageing and longevity database enrichment analysis

Lists of genes previously associated with longevity, post-mitotic cell maintenance, genes with age-dependent expression and genes associated with other molecular functions such as DNA repair, autophagy, immunity and inflammation, oxidative stress, epigenetic and apoptosis considered necessary for the regulation of ageing and longevity (Supplementary Table 3) were downloaded from various sources.

Gene numbers associated with each database are shown in Supplementary Table 3). DNA repair genes^72^ were examined as DNA damage has been suggested to play an essential role in ageing^113^. In several studies, it has been found that long-lived organisms had increased DNA repair activity^114–117^. Inflammation is a natural immune response to injury or infection. However, chronic inflammation can damage cells and tissues, which is thought to play a role in ageing^65^. Oxidative stress is caused by an imbalance between the production of reactive oxygen species (ROS) and the body’s ability to detoxify them. ROS can damage biomolecules, including DNA, and they are thought to play a role in ageing^67^. Various factors, including ageing, environmental toxins, and lifestyle choices, can induce epigenetic markers in DNA and histone proteins. Epigenetic changes can contribute to ageing by altering gene expression in cell growth, metabolism, and repair^68,118^. Notably, specific categories of epigenetic data possess the capacity to exert a transgenerational impact on the longevity of progeny. These investigations yield several pivotal insights: instead of being genetically preordained, our life expectancy is predominantly under the sway of epigenetic determinants; dietary and other environmental factors have the potential to modify our life span by altering epigenetic data; and inhibitors targeting epigenetic enzymes can exert influence over the life expectancy of model organisms. These novel discoveries enhance our comprehension of the mechanisms underpinning the ageing process. Given the reversible nature of epigenetic data, these inquiries illuminate promising avenues for therapeutic interventions in ageing and age-related ailments, including cancer^118^. Apoptosis-related genes^76^ were examined as apoptosis can delay ageing by removing damaged and senescent cells from tissue, but it can promote ageing when it eliminates irreplaceable post-mitotic cells^95,119^. Autophagy is a cellular process that degrades damaged or unnecessary cellular components. Autophagy decreases with age, contributing to the accumulation of cellular debris and the development of age-related diseases^66^.

Secondly, we examined the age-dependent expressed genes. Genes with age-dependent gene expression increased or decreased their activity in an age-dependent manner and were compiled from two different studies among MLSP-associated genes^27,77^. Genes with age-dependent transcription profiles may be markers of the ageing process and/or parts of the molecular mechanism to counteract the deleterious effects of the ageing process^27,77^.

Third, we examined two manually curated datasets—cell senescence-promoting genes and longevity-associated genes. Cell senescence-promoting genes^29^ are thought to contribute to organism ageing. Longevity-associated genes are a database of ageing or longevity-related genes in humans and model organisms. This database is divided into pro and anti-longevity genes depending on whether they have been deemed to promote or hinder longevity^78,79^.

Fourth, we also examined longevity-modifying interventions. Dietary restriction benefit suppressor genes counteract the life-extending effects of caloric restriction^80^. It has been suggested that dietary restriction can boost longevity by reducing the intake of nutrients and delaying age-related degeneration^120–122^. Life-extending drug target genes were compiled from several studies^81–84^.

Finally, we examined genes associated with lifespan from several studies. “Human longevity-associated variants” is a dataset compiled from manually curated literature of cross-sectional and extreme longevity (centenarians) GWAS studies in healthy individuals^85^. Only genes with variants significantly associated with longevity in at least one human population were selected^85^. Longevity protein evolution genes were identified as those with a higher protein evolution rate associated with a longer species MLSP^123^). Maximum lifespan genes are an independent database of MLSP-associated genes identified from transcriptome comparative analysis in 26 mammalian species with varying MLSP^86^. Post-mitotic cell maintenance genes are those whose expression patterns correlate with the longevity of different cell types in a non-dividing state. These are assumed to be necessary for overall organism longevity^25,26^. Chi-squared tests were applied to assess if genes in each gene list were over or underrepresented among MLSP-associated genes. For those gene lists with an available background gene set, we restricted the analysis to genes present in both the latter background and our own background gene set of 4121 gene families encompassing 10,235 genes.

GO enrichment analysis was performed by comparing gene sets from previous studies at the functional level, using the background and focal gene sets specified in Supplementary Table 2. For datasets lacking background genes, we used the MLSP-associated genes background. The analysis followed the same framework as the gene ontology term enrichment analysis, except that GO terms annotated to fewer than 200 genes were excluded.

### Alternative splicing analysis

Data for alternative splicing, comprising the number of unique transcripts and expression level for each human protein-coding gene, was obtained from the MeDAS database^124^. Non-parametric tests (Wilcoxon rank sum test) were then used to assess differences in alternative splicing and gene expression in trait associated genes.

## Electronic supplementary material

Below is the link to the electronic supplementary material.

Supplementary Material 1

Supplementary Material 2

Supplementary Material 3

Supplementary Material 4

## Data Availability

The datasets used and/or analysed during the current study available from the corresponding author on reasonable request.
