# Supplementary material for: Maximum lifespan and brain size in mammals are associated with gene family size expansion related to immune system functions
Source: Sci Rep. 2025 Apr 29;15:15087. doi: 10.1038/s41598-025-98786-3 (PMC12041557; doi:10.1038/s41598-025-98786-3)
Supplement: Supplementary file 1 — Supplementary Material 1 [file 41598_2025_98786_MOESM1_ESM.pdf]

Supplementary data 1: **Maximum lifespan and brain size in mammals are associated with gene family size expansion related to immune system functions**

Effect size per species of LOO vs original

Effect Size Interpretation (Cohen's d Scale)

**Cohen's d**	**Effect size**	**Interpretation**
0 - 0.2	Negligible	The groups are very similar
0.2 - 0.5	Small	Small difference, but possibly meaningful
0.5 - 0.8	Medium	Moderate difference
> 0.8	Large	Strong difference

Effect size using coef t values and significant p-values. The Wilcox on R scores

Wilcox-test of significant PGLS results from LOO analysis for Ailuropoda_melanoleuca
Cohen test for effect size Estimate 0.0385302 | Conf.int: -0.07900151 0.1560619 | Magnitude: 1

Wilcox-test of significant PGLS results from LOO analysis for Bos_taurus
Cohen test for effect size Estimate 0.005101032 | Conf.int: -0.1122631 0.1224652 | Magnitude: 1

Wilcox-test of significant PGLS results from LOO analysis for Callithrix_jacchus
Cohen test for effect size Estimate 0.0228372 | Conf.int: -0.09366864 0.139343 | Magnitude: 1

Wilcox-test of significant PGLS results from LOO analysis for Canis_familiaris
Cohen test for effect size Estimate -0.03926579 | Conf.int: -0.1567455 0.07821389 | Magnitude: 1

Wilcox-test of significant PGLS results from LOO analysis for Capra_hircus
Cohen test for effect size Estimate 0.001595158 | Conf.int: -0.1157168 0.1189072 | Magnitude: 1

Wilcox-test of significant PGLS results from LOO analysis for Cavia_porcellus
Cohen test for effect size Estimate -0.01500894 | Conf.int: -0.1322191 0.1022013 | Magnitude: 1

Wilcox-test of significant PGLS results from LOO analysis for Cebus_capucinus_imitator
Cohen test for effect size Estimate 0.0668477 | Conf.int: -0.04993578 0.1836312 | Magnitude: 1

Wilcox-test of significant PGLS results from LOO analysis for Chinchilla_lanigera
Cohen test for effect size Estimate -0.01853068 | Conf.int: -0.1363722 0.09931083 | Magnitude: 1

Wilcox-test of significant PGLS results from LOO analysis for Colobus_angolensis_palliatus
Cohen test for effect size Estimate -0.001851755 | Conf.int: -0.1194252 0.1157217 | Magnitude: 1

Wilcox-test of significant PGLS results from LOO analysis for Dasypus_novemcinctus
Cohen test for effect size Estimate -0.02708976 | Conf.int: -0.1450416 0.09086204 | Magnitude: 1

Wilcox-test of significant PGLS results from LOO analysis for Equus_asinus_asinus
Cohen test for effect size Estimate 0.02106148 | Conf.int: -0.09594534 0.1380683 | Magnitude: 1

Wilcox-test of significant PGLS results from LOO analysis for Equus_caballus
Cohen test for effect size Estimate 0.01920557 | Conf.int: -0.09774986 0.136161 | Magnitude: 1

Wilcox-test of significant PGLS results from LOO analysis for Felis_catus
Cohen test for effect size Estimate 0.001536026 | Conf.int: -0.1158801 0.1189521 | Magnitude: 1

Wilcox-test of significant PGLS results from LOO analysis for Gorilla_gorilla
Cohen test for effect size Estimate 0.1280602 | Conf.int: 0.008705731 0.2474148 | Magnitude: 1

Wilcox-test of significant PGLS results from LOO analysis for Heterocephalus_glaber
Cohen test for effect size Estimate -0.2195507 | Conf.int: -0.3371118 -0.1019896 | Magnitude: 2

Wilcox-test of significant PGLS results from LOO analysis for Homo_sapiens
Cohen test for effect size Estimate -0.5878973 | Conf.int: -0.8063134 -0.3694812 | Magnitude: 3

Wilcox-test of significant PGLS results from LOO analysis for Ictidomys_tridecemlineatus
Cohen test for effect size Estimate -0.02887118 | Conf.int: -0.1462412 0.08849888 | Magnitude: 1

Wilcox-test of significant PGLS results from LOO analysis for Loxodonta_africana
Cohen test for effect size Estimate 0.1324311 | Conf.int: 0.01565246 0.2492098 | Magnitude: 1

Wilcox-test of significant PGLS results from LOO analysis for Macaca_fascicularis
Cohen test for effect size Estimate -0.1012693 | Conf.int: -0.2163373 0.01379871 | Magnitude: 1

Wilcox-test of significant PGLS results from LOO analysis for Macaca_mulatta
Cohen test for effect size Estimate -0.08274338 | Conf.int: -0.1967439 0.03125718 | Magnitude: 1

Wilcox-test of significant PGLS results from LOO analysis for Macaca_nemestrina
Cohen test for effect size Estimate -0.00476462 | Conf.int: -0.1214656 0.1119364 | Magnitude: 1

Wilcox-test of significant PGLS results from LOO analysis for Meriones_unguiculatus
Cohen test for effect size Estimate 0.01464857 | Conf.int: -0.1028738 0.132171 | Magnitude: 1

Wilcox-test of significant PGLS results from LOO analysis for Microcebus_murinus
Cohen test for effect size Estimate 0.004058023 | Conf.int: -0.1134105 0.1215265 | Magnitude: 1

Wilcox-test of significant PGLS results from LOO analysis for Microtus_ochrogaster
Cohen test for effect size Estimate -0.008284462 | Conf.int: -0.1258058 0.1092368 | Magnitude: 1

Wilcox-test of significant PGLS results from LOO analysis for Monodelphis_domestica
Cohen test for effect size Estimate 0.06952949 | Conf.int: -0.04637781 0.1854368 | Magnitude: 1

Wilcox-test of significant PGLS results from LOO analysis for Mus_musculus
Cohen test for effect size Estimate 0.01717315 | Conf.int: -0.09943013 0.1337764 | Magnitude: 1

Wilcox-test of significant PGLS results from LOO analysis for Mustela_putorius_furo
Cohen test for effect size Estimate -0.007058173 | Conf.int: -0.1242671 0.1101507 | Magnitude: 1

Wilcox-test of significant PGLS results from LOO analysis for Myotis_lucifugus
Cohen test for effect size Estimate -0.1183778 | Conf.int: -0.2358445 -0.0009111009 | Magnitude: 1

Wilcox-test of significant PGLS results from LOO analysis for Neovison_vison
Cohen test for effect size Estimate 0.001688101 | Conf.int: -0.1158853 0.1192615 | Magnitude: 1

Wilcox-test of significant PGLS results from LOO analysis for Nomascus_leucogenys
Cohen test for effect size Estimate -0.05324107 | Conf.int: -0.1712084 0.06472622 | Magnitude: 1

Wilcox-test of significant PGLS results from LOO analysis for Octodon_degus
Cohen test for effect size Estimate 0.003164963 | Conf.int: -0.1146741 0.121004 | Magnitude: 1

Wilcox-test of significant PGLS results from LOO analysis for Oryctolagus_cuniculus
Cohen test for effect size Estimate -0.002818577 | Conf.int: -0.120287 0.1146498 | Magnitude: 1

Wilcox-test of significant PGLS results from LOO analysis for Otolemur_garnettii
Cohen test for effect size Estimate 0.004808883 | Conf.int: -0.1132458 0.1228635 | Magnitude: 1

Wilcox-test of significant PGLS results from LOO analysis for Ovis_aries
Cohen test for effect size Estimate -0.005842067 | Conf.int: -0.1231543 0.1114702 | Magnitude: 1

Wilcox-test of significant PGLS results from LOO analysis for Pan_paniscus
Cohen test for effect size Estimate -0.1653905 | Conf.int: -0.2817487 -0.04903232 | Magnitude: 1

Wilcox-test of significant PGLS results from LOO analysis for Pan_troglodytes
Cohen test for effect size Estimate 0.03248001 | Conf.int: -0.08330521 0.1482652 | Magnitude: 1

Wilcox-test of significant PGLS results from LOO analysis for Panthera_pardus
Cohen test for effect size Estimate -0.003892231 | Conf.int: -0.1214131 0.1136287 | Magnitude: 1

Wilcox-test of significant PGLS results from LOO analysis for Papio_anubis
Cohen test for effect size Estimate -0.0302739 | Conf.int: -0.1476967 0.0871489 | Magnitude: 1

Wilcox-test of significant PGLS results from LOO analysis for Peromyscus_maniculatus_bairdii
Cohen test for effect size Estimate -0.00580254 | Conf.int: -0.1235883 0.1119832 | Magnitude: 1

Wilcox-test of significant PGLS results from LOO analysis for Phascolarctos_cinereus
Cohen test for effect size Estimate 0.01066686 | Conf.int: -0.1064397 0.1277734 | Magnitude: 1

Wilcox-test of significant PGLS results from LOO analysis for Rattus_norvegicus
Cohen test for effect size Estimate 0.03778908 | Conf.int: -0.07932712 0.1549053 | Magnitude: 1

Wilcox-test of significant PGLS results from LOO analysis for Saimiri_boliviensis_boliviensis
Cohen test for effect size Estimate -0.01155753 | Conf.int: -0.1291319 0.1060169 | Magnitude: 1

Wilcox-test of significant PGLS results from LOO analysis for Sus_scrofa
Cohen test for effect size Estimate 0.005730176 | Conf.int: -0.1121091 0.1235694 | Magnitude: 1

Wilcox-test of significant PGLS results from LOO analysis for Tursiops_truncatus
Cohen test for effect size Estimate 0.02040872 | Conf.int: -0.09599786 0.1368153 | Magnitude: 1

Wilcox-test of significant PGLS results from LOO analysis for Vombatus_ursinus
Cohen test for effect size Estimate -0.01423878 | Conf.int: -0.1317086 0.1032311 | Magnitude: 1

Wilcox-test of significant PGLS results from LOO analysis for Vulpes_vulpes
Cohen test for effect size Estimate -0.03497416 | Conf.int: -0.1524515 0.08250318 | Magnitude: 1

Wilcox test of statistically significant Rs coming from the t-value test.

Ailuropoda_melanoleuca has a NOT significant p-value of 0.2500087

Bos_taurus has a NOT significant p-value of 0.9956395

Callithrix_jacchus has a NOT significant p-value of 0.2678731

Canis_familiaris has a NOT significant p-value of 0.1605927

Capra_hircus has a NOT significant p-value of 0.9704099

Cavia_porcellus has a NOT significant p-value of 0.9551943

Cebus_capucinus_imitator has a significant p-value of 0.01585713

Chinchilla_lanigera has a NOT significant p-value of 0.4498999

Colobus_angolensis_palliatus has a NOT significant p-value of 0.8349771

Dasypus_novemcinctus has a NOT significant p-value of 0.459551

Equus_asinus_asinus has a NOT significant p-value of 0.6066091

Equus_caballus has a NOT significant p-value of 0.2921118

Felis_catus has a NOT significant p-value of 0.7783253

Gorilla_gorilla has a significant p-value of 0.0003191049

Heterocephalus_glaber has a significant p-value of 3.417087e-08

Homo_sapiens has a NOT significant p-value of 0.1621582

Ictidomys_tridecemlineatus has a NOT significant p-value of 0.6307684

Loxodonta_africana has a significant p-value of 0.0002204717

Macaca_fascicularis has a significant p-value of 0.02962121

Macaca_mulatta has a NOT significant p-value of 0.1011675

Macaca_nemestrina has a NOT significant p-value of 0.7310688

Meriones_unguiculatus has a NOT significant p-value of 0.5470535

Microcebus_murinus has a NOT significant p-value of 0.8232496

Microtus_ochrogaster has a NOT significant p-value of 0.6707585

Monodelphis_domestica has a significant p-value of 0.01410006

Mus_musculus has a NOT significant p-value of 0.345189

Mustela_putorius_furo has a NOT significant p-value of 0.8973031

Myotis_lucifugus has a significant p-value of 0.0002681611

Neovison_vison has a NOT significant p-value of 0.5666422

Nomascus_leucogenys has a NOT significant p-value of 0.06187975

Octodon_degus has a NOT significant p-value of 0.7125981

Oryctolagus_cuniculus has a NOT significant p-value of 0.7412678

Otolemur_garnettii has a NOT significant p-value of 0.8109843

Ovis_aries has a NOT significant p-value of 0.5992237

Pan_paniscus has a significant p-value of 0.0001356961

Pan_troglodytes has a NOT significant p-value of 0.2625003

Panthera_pardus has a NOT significant p-value of 0.499262

Papio_anubis has a NOT significant p-value of 0.2881053

Peromyscus_maniculatus_bairdii has a NOT significant p-value of 0.9649743

Phascolarctos_cinereus has a NOT significant p-value of 0.7119419

Rattus_norvegicus has a NOT significant p-value of 0.2482993

Saimiri_boliviensis_boliviensis has a NOT significant p-value of 0.7274119

Sus_scrofa has a NOT significant p-value of 0.8175211

Tursiops_truncatus has a NOT significant p-value of 0.9840717

Vombatus_ursinus has a NOT significant p-value of 0.9417315

Vulpes_vulpes has a NOT significant p-value of 0.11097
