# Supplementary material for: Maximum lifespan and brain size in mammals are associated with gene family size expansion related to immune system functions
Source: Sci Rep. 2025 Apr 29;15:15087. doi: 10.1038/s41598-025-98786-3 (PMC12041557; doi:10.1038/s41598-025-98786-3)
Supplement: Supplementary file 3 — Supplementary Material 3 [file 41598_2025_98786_MOESM3_ESM.pdf]

ReadMe file of supplementary data: **Maximum lifespan and brain size in mammals are associated with gene family size expansion related to immune system functions**

Author: Huseyin Kilili and Benjamin Padilla-Morales
Affiliation: University of Bath
Email: hk611@bath.ac.uk, benjamin.padilla.morales@bath.edu

Overview

This research article investigates the relationship between gene family size expansions and the evolution of maximum lifespan potential (MLSP) in mammals. The study focuses on 46 fully sequenced mammalian species and examines the association between gene family size and MLSP, taking into account potential confounding factors like body mass, relative brain size, gestation time, and age at sexual maturity. The researchers discovered a significant association between gene family expansion and MLSP, particularly in gene families enriched for immune system functions. Moreover, the study found that relative brain size, but not body mass or other life history traits, significantly influences gene family expansion associated with MLSP. The findings suggest a shared evolutionary pathway between longer lifespans and larger brains in mammals, possibly driven by expansions in immune-related gene families. The study concludes that gene family expansions have likely contributed to the evolution of longer lifespans in mammals, emphasizing the importance of considering relative brain size in future studies examining the genomic signatures of MLSP.

Contents

Supporting information listing

Supplementary Figure 1. Pairwise correlations among maximum lifespan potential, relative brain size, body mass, gestation time and age at sexual maturity in 46 mammalian species. Pairwise correlations between five key traits: (A) Maximum lifespan potential (MLSP), (B) relative brain size, (C) body mass, (D) gestation time, and (E) age at sexual maturity, across 46 mammalian species with fully sequenced genomes. The upper triangle of the matrix displays the Pearson correlation coefficients with associated p-values in parentheses. The lower triangle contains scatter plots with fitted regression lines for each trait pair, illustrating the linear relationships. Diagonal panels represent the distribution density plots for each trait.

Supplementary Figure 2. Significant associations of biological functional annotations overlaped between longevity studies. GO Enrichment analysis of longevity-related databases and MLSP-associated genes. Database gene association abbreviations: AgeCellExp_Neg (Age-dependent Cellular Expression - Negative), AgeCellExp_Pos (Age-dependent Cellular Expression - Positive), Turnover_Neg (Cellular Turnover - Negative), Turnover_Pos (Cellular Turnover - Positive), AgeExp_Neg (MLSP-Ei Age-dependent Expression - Negative), AgeExp_Pos (MLSP-Ei Age-dependent Expression - Positive), Apoptosis (Apoptosis), Senescence (Cell Senescence Promoters), DNA_Repair (DNA Repair), Diet_Resist (Dietary Restriction Benefit Suppressors), Ei_Assoc (Ei-associated), Longevity_Var (Human Longevity Associated Variants), LifeExt_Drugs (Life-Extending Drug Targets), MLSP_Assoc (MLSP-associated), PM_Longevity (Post-mitotic Cell Longevity). Colour intensity increases for more significnat p-values.

Supplementary Table 1. Trait and data sources for 46 mammalian species. Note: Table with traits used in this study for 46 mammalian species and corresponding references.

Supplementary Table 2. Contracting gene families associated with MLSP and relative brain size. Note: List of gene families associated with MLSP and relative brain size analysis and corresponding statistics.

Supplementary Table 3. Lists of genes associated with MLSP and longevity-related processes. Note: MLSP associated genes and their representation across all databases

Supplementary Table 4. Names, sources, a priori expectations, and enrichment results for longevity-associated databases. Note: List of MLSP related databased with their description and representation expectations.

For any questions or inquiries, please contact Huseyin Kilili or Benjamin Padilla-Morales at the provided email address.
