# Supplementary material for: Maximum lifespan and brain size in mammals are associated with gene family size expansion related to immune system functions
Source: Sci Rep. 2025 Apr 29;15:15087. doi: 10.1038/s41598-025-98786-3 (PMC12041557; doi:10.1038/s41598-025-98786-3)
Supplement: Supplementary file 4 — Supplementary Material 4 [file 41598_2025_98786_MOESM4_ESM.pdf]

Supplementary Figures: **Maximum lifespan and brain size in mammals are associated with gene family size expansion related to immune system functions**

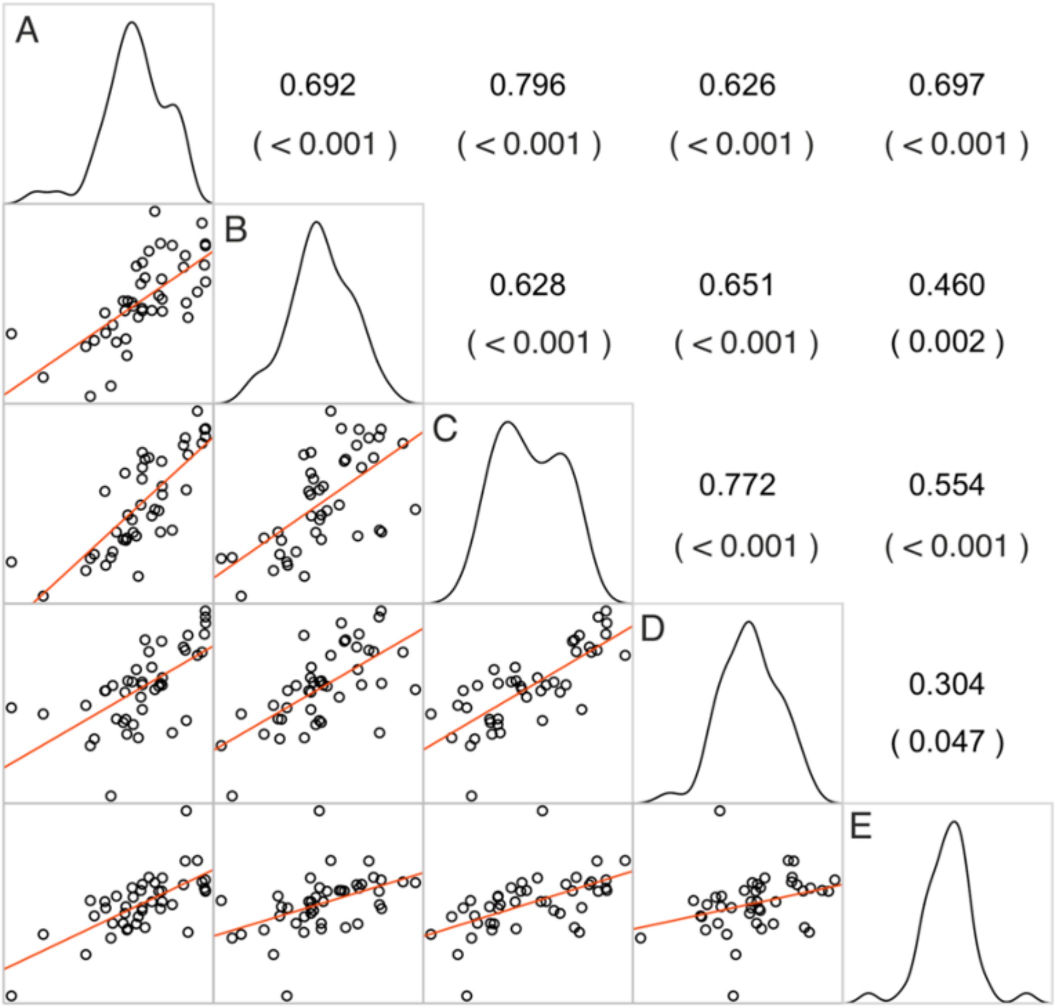

Supplementary Figure 1. Pairwise correlations among maximum lifespan potential, relative brain size, body mass, gestation time and age at sexual maturity in 46 mammalian species. Pairwise correlations between five key traits: (A) Maximum lifespan potential (MLSP), (B) relative brain size, (C) body mass, (D) gestation time, and (E) age at sexual maturity, across 46 mammalian species with fully sequenced genomes. The upper triangle of the matrix displays the Pearson correlation coefficients with associated p-values in parentheses. The lower triangle contains scatter plots with fitted regression lines for each trait pair, illustrating the linear relationships. Diagonal panels represent the distribution density plots for each trait.

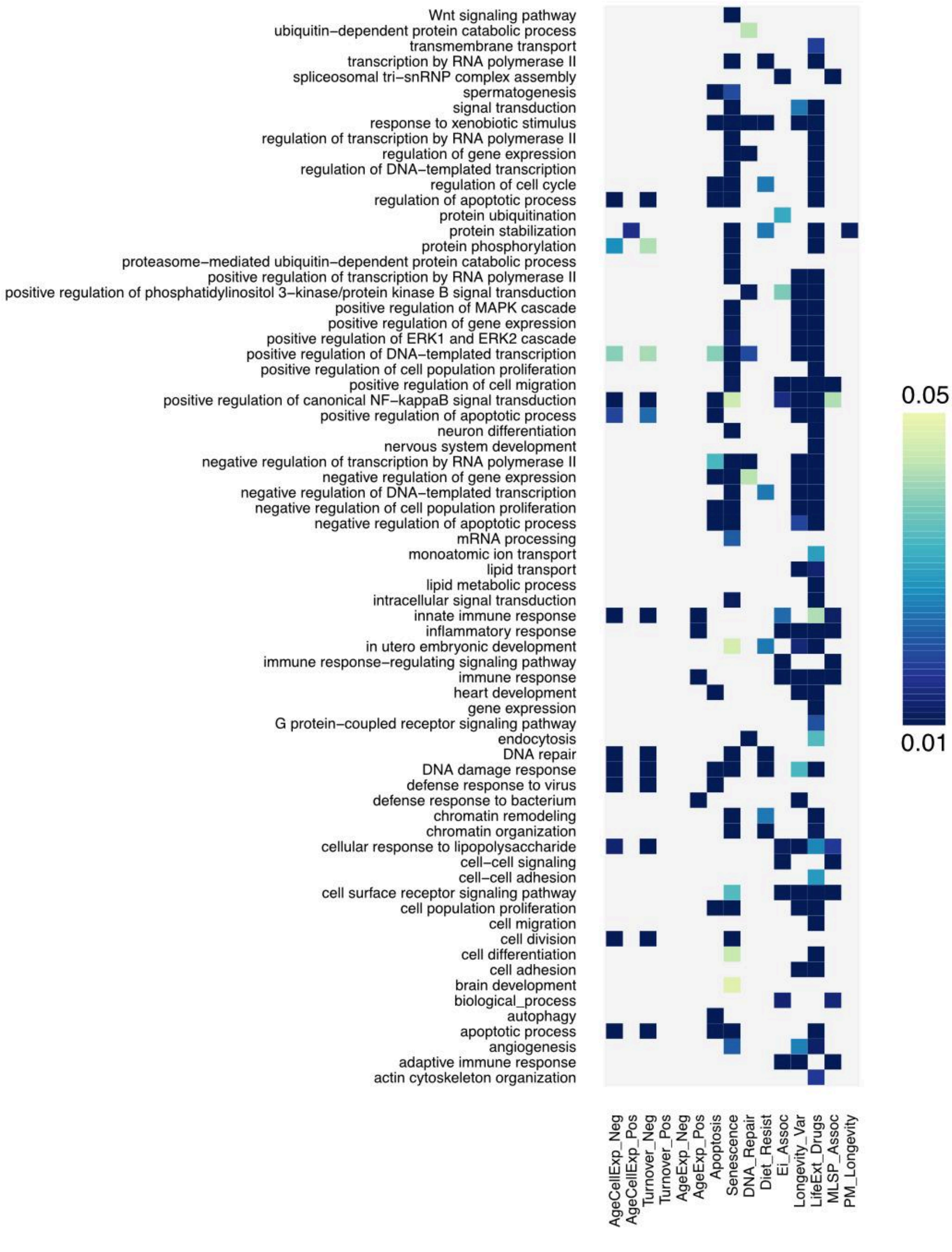

Supplementary Figure 2. Significant associations of biological functional annotations overlaped between longevity studies. GO Enrichment analysis of longevity-related databases and MLSP-associated genes. Database gene association abbreviations: AgeCellExp_Neg (Age-dependent Cellular Expression -

Negative), AgeCellExp_Pos (Age-dependent Cellular Expression - Positive), Turnover_Neg (Cellular Turnover - Negative), Turnover_Pos (Cellular Turnover - Positive), AgeExp_Neg (MLSP-Ei Age-dependent Expression - Negative), AgeExp_Pos (MLSP-Ei Age-dependent Expression - Positive), Apoptosis (Apoptosis), Senescence (Cell Senescence Promoters), DNA_Repair (DNA Repair), Diet_Resist (Dietary Restriction Benefit Suppressors), Ei_Assoc (Ei-associated), Longevity_Var (Human Longevity Associated Variants), LifeExt_Drugs (Life-Extending Drug Targets), MLSP_Assoc (MLSP-associated), PM_Longevity (Post-mitotic Cell Longevity). Colour intensity increases for more significnat *p-values*.
